# Condomless sex in HIV-diagnosed men who have sex with men in the UK: prevalence, correlates, and implications for HIV transmission

**DOI:** 10.1136/sextrans-2016-053029

**Published:** 2017-07-05

**Authors:** Marina Daskalopoulou, Alison J Rodger, Andrew N Phillips, Lorraine Sherr, Jonathan Elford, Jeffrey McDonnell, Simon Edwards, Nicky Perry, Ed Wilkins, Simon Collins, Anne M Johnson, William J Burman, Andrew Speakman, Fiona C Lampe

**Affiliations:** 1 Research Department of Infection and Population Health, University College London, London, UK; 2 City University London, London, UK; 3 Mortimer Market Centre, Central and North West London NHS Foundation Trust, London, UK; 4 Brighton and Sussex University Hospitals NHS Trust, Brighton, UK; 5 Pennine Acute Hospitals NHS Trust, Manchester, UK; 6 HIV i-Base, London, UK; 7 Denver Public Health, Denver, Colorado, USA

**Keywords:** GAY MEN, HIV, SEXUAL BEHAVIOUR, EPIDEMIOLOGY (GENERAL), DRUG MISUSE

## Abstract

**Objective:**

HIV transmission is ongoing among men who have sex with men (MSM) in the UK. Sex without a condom (condomless sex, CLS) is the main risk factor. We investigated the prevalence of and factors associated with types of CLS.

**Methods:**

Cross-sectional questionnaire study in UK HIV clinics in 2011/2012 (ASTRA). MSM diagnosed with HIV for ≥3 months reported on anal and vaginal sex, CLS with HIV-serodifferent partners (CLS-D) and CLS with HIV-seroconcordant (CLS-C) partners in the previous 3 months. Mutually exclusive sexual behaviours were as follows: (1) Higher HIV risk CLS-D (not on antiretroviral therapy (ART) or clinic-recorded viral load(VL) >50 c/mL), (2) Other CLS-D, (3) CLS-C without CLS-D, (4) Condom-protected sex only and (5) No anal or vaginal sex. Associations were examined of sociodemographic, HIV-related, lifestyle, and other sexual measures with the five categories of sexual behaviour. We examined the prevalence of higher HIV risk CLS-D incorporating (in addition to ART and VL) time on ART, ART non-adherence, and recent sexually transmitted infections (STIs).

**Results:**

Among 2189 HIV-diagnosed MSM (87% on ART), prevalence of any CLS in the past 3 months was 38.2% (95% CI 36.2% to 40.4%) and that of any CLS-D was 16.3% (14.8%–17.9%). The five-category classification was as follows: (1) Higher HIV risk CLS-D: 4.2% (3.5% to 5.2%), (2) Other CLS-D: 12.1% (10.8% to 13.5%), (3) CLS-C without CLS-D: 21.9% (20.2% to 23.7%), (4) Condom-protected sex only: 25.4% (23.6% to 27.3%) and (5) No anal or vaginal sex: 36.4% (34.3% to 38.4%). Compared with men who reported condom-protected sex only, MSM who reported any CLS in the past 3 months had higher prevalence of STIs, chemsex-associated drug use, group sex, higher partner numbers, and lifetime hepatitis C. Prevalence of higher HIV risk CLS-D ranged from 4.2% to 7.5% according to criteria included.

**Conclusion:**

CLS was prevalent among HIV-diagnosed MSM, but CLS-D with higher HIV transmission risk was overall low. CLS-D is no longer the most appropriate measure of HIV transmission risk behaviour among people with diagnosed HIV; accounting for VL is important.

## Introduction

HIV transmission is ongoing among men who have sex with men (MSM) in the UK, despite continfigued prevention efforts, universal access to HIV care, high antiretroviral therapy (ART) uptake and high levels of HIV plasma viral load (VL) suppression in the diagnosed population.^1^ HIV incidence is driven by patterns of sexual risk behaviour among HIV-diagnosed, HIV-undiagnosed, and HIV-negative MSM.^2^ Given the increase in uptake of HIV testing and ART use in the past decade, sustained HIV incidence among MSM points to increasing sexual risk behaviours during this period.^3^ The majority of HIV transmissions among MSM in the UK derive from men with as yet undiagnosed HIV.^4 5^ However, modelling studies estimate that sizeable proportions of transmissions originate from HIV-diagnosed individuals (estimated 18% in the UK^4^ and 29% in the Netherlands^6^). Over the past decade, diagnoses of other STIs, such as gonorrhoea, syphilis, and genital herpes, have also increased considerably among MSM in the UK, and are prevalent among HIV-diagnosed MSM.^7^

Among HIV-diagnosed people, the primary indicator of HIV transmission risk is anal or vaginal sex without a condom (condomless sex, CLS), in particular, with partners who are HIV-negative or do not know their HIV serostatus (CLS with HIV-serodifferent partners, CLS-D). The concept of ‘high risk sex’ has evolved substantially over the past two decades: first, with the introduction of combination ART for the treatment of HIV (1995/1996), and second, with the high-publicity ‘Swiss Statement’ (2008) that asserted (with caveats) that an HIV-positive person on ART with viral suppression is not sexually infectious^8^ and, subsequently, with increasing evidence from observational studies and randomised controlled trials on the crucial role of HIV VL suppression on reducing HIV transmission.^9–11^ There is now concrete evidence that the risk of HIV transmission during CLS-D among MSM is extremely low when the HIV-positive partner is on virally suppressive ART.^12 13^ Results from recent trials (since 2012) have also shown the substantial protective effect conferred by oral pre-exposure prophylaxis (PrEP) in reducing the risk of HIV acquisition among HIV-negative individuals.^14–16^ As a result, the concept of high-risk sex will further evolve with additional evidence and use of PrEP. CLS-D may no longer be the most appropriate measure of higher risk CLS in the context of HIV transmission for HIV-diagnosed individuals. Understanding the prevalence and drivers of various types of CLS among MSM living with HIV warrants further study and is important in informing clinical care and HIV/STI prevention strategies.

Few studies have assessed the prevalence of different types of CLS among representative samples of HIV-diagnosed MSM in the UK or examined CLS with an appreciable risk of HIV transmission (accounting for ART use and VL level). This study describes the prevalence and factors associated with recent CLS among HIV-diagnosed MSM in the UK, including CLS with HIV-serodifferent partners that could confer higher risk of HIV transmission, and other CLS with HIV-serodifferent and HIV-seroconcordant partners.

## Methods

### ASTRA study

The Antiretrovirals, Sexual Transmission Risk and Attitudes (ASTRA) study recruited HIV-diagnosed adults attending eight hospital HIV outpatient clinics in the UK from 2011 to 2012.^2^ Participants completed a confidential, self-administered questionnaire that sought information on sociodemographic, HIV-related, health and lifestyle factors, as well as sexual behaviours. Consent to participate included permission to collect latest CD4 count and HIV plasma VL from clinic records (latest value available to the participant). Current ART use, date of starting ART, and ART non-adherence in the past 3 months were defined according to self-report on the questionnaire. Men who identified as gay or bisexual or who reported sex with men in the previous three months were classified as MSM. Participants were asked about the use of any of the following recreational drugs in the past 3 months: acid/LSD/magic mushrooms, anabolic steroids, cannabis, cocaine, crack cocaine, codeine, crystal methamphetamine, ecstasy (MDMA), GHB/GBL (liquid ecstasy), heroin, ketamine, khat (chat), mephedrone, morphine, opium, poppers (nitrites), speed (amphetamine) and erectile dysfunction drugs (such as Viagra, Cialis and similar pro-erection drugs). Polydrug use was defined as the use of ≥4 drugs in the previous three months. Chemsex-associated drug use was defined as the use of one or more of the following: mephedrone, GHB/GBL, and crystal methamphetamine.^17^ Social support was assessed using a modified version of the Duke-UNC Functional Social Support Questionnaire.^18^ Harmful alcohol consumption (that increases the risk of harmful consequences to the user or others) was defined as a score of ≥6 on a modified version of the WHO AUDIT-C (first two questions only).^19^

### Sexual behaviours

The questionnaire enquired about the following sexual behaviours in the previous 3 months: (1) any anal or vaginal sex, (2) any CLS (anal or vaginal sex without a condom), (3) any CLS with HIV-serodifferent partners (CLS-D, CLS with a man or woman who did not have HIV or whose HIV status the participant did not know) and (4) any CLS with HIV-seroconcordant partners (CLS-C, CLS with a man or woman the participant knew also had HIV). These four measures provided the overall estimates of any sex, any CLS, any CLS-D, and any CLS-C in the past 3 months.

Using these four variables and with additional information on ART use and clinic VL level, it was possible to then classify each participant into one of five mutually exclusive categories of sexual behaviour in the past 3 months. The categories are presented below:table 1

**Table 1:** 

Sexual behaviour category in the past 3 months (mutually exclusive)	Definition
1. Higher HIV risk CLS-D	CLS with HIV-negative or HIV-unknown serostatus partners (CLS-D) and either: not on ART at the time of the questionnaire or has latest clinic VL >50 c/mL. Includes n=8 participants who reported having CLS without specifying their partner(s) HIV serostatus
2. Other CLS-D	Any other CLS with HIV-serodifferent partners (but not CLS-D higher HIV risk). Includes n=23 participants who reported having CLS without specifying their partner(s) HIV serostatus
3. ‘CLS-C without CLS-D’	CLS with HIV-seroconcordant (other HIV-positive) partners only (CLS-C), with no CLS-D partners
4. Condom-protected sex	Condom-protected sex only
5. No anal or vaginal sex	

ART, antiretroviral therapy; CLS, condomless sex; N=31 participants with ’possible ClS-D' (reported having CLS but did not specify partner(s) serostatus) were included as CLS-D in this classification^20^.

Therefore, according to this five-category variable, a man who had CLS with other HIV-positive men, but not with HIV-negative or HIV-unknown status men, was classified as ‘CL^20^S-C without CLS-D’ (category 3); a man who reported anal or vaginal sex but did not report either CLS-D or CLS-C was classified as having condom-protected sex only (category 4).

### Other sexual behaviours, STIs and attitudes

Self-reported STI diagnosis in the past 3 months included syphilis, gonorrhoea, chlamydia, lymphogranuloma venereum (LGV), new hepatitis B or C, new or recurrent genital herpes or warts, trichomonas, and non-specific urethritis/non-gonococcal urethritis (NSU/NGU). Participants were asked to report the total number of partners (men and women) they had sex with in the past 3 months, the total number of HIV-negative or HIV-unknown serostatus partners they had sex with without a condom in the previous 3 months (CLS-D partners) and the total number of HIV-positive partners participants had sex with without a condom in the previous 3 months (CLS-C partners). In the past 3 months, the following were also assessed: group sex (with more than one other person on the same occasion) and use of the internet to find sex. Participants rated their agreement to statements on condom use and HIV transmission on a 5-level Likert scale (from ‘strongly agree’ to ‘strongly disagree’). These statements were as follows: (1) difficulty negotiating condom use (‘I find it difficult to discuss condom use with a new sexual partner’), (2) lower condom use with casual partners (‘I am less likely to use a condom with a casual partner’) and (3) worry about HIV transmission (‘I am worried that I could have infected someone else with HIV in the past few months’). Responses rated ‘strongly agree’ and ‘tend to agree’ were combined into one category (versus ‘undecided’, ‘tend to disagree’ and ‘strongly disagree’). Participants also reported the total number of new sexual partners in the past year and whether they had ever received a diagnosis of hepatitis C. Sexual positioning (insertive or receptive partner) and withdrawal before ejaculation were assessed among MSM who had CLS-D in the past 3 months only.

### Additional definitions of higher HIV-risk CLS-D

We sought to assess alternative definitions of higher HIV risk CLS with HIV-serodifferent partners (CLS-D) by incorporating factors additional to ART use and VL level. Factors were selected on the basis of any evidence that these may compromise low HIV infectiousness even if the participant was on ART with last recorded VL ≤50 c/mL. Self-reported factors (in addition to reporting CLS-D and either not being on ART or having latest clinic VL ≥50 c/mL) included the following: (1) started ART ≤9 months ago, (2) ART non-adherence (missing ≥2 consecutive days of ART on ≥2 occasions in past 3 months) and (3) diagnosis of STI (other than HIV) in the past 3 months.

### Statistical methods

#### Exclusion of recently diagnosed participants

To improve validity of sexual behaviour questions with a 3-month recall, we excluded MSM who were diagnosed three or fewer months prior to the date of the questionnaire completion (n=59).

### Prevalence of sexual behaviours

Among all MSM, the prevalence (and 95% CI) of any sex, any CLS, any CLS-D, and any CLS-C was assessed in the previous 3 months. The prevalence of specific sexual behaviours in the past 3 months was then assessed according to the mutually exclusive five-category variable.

Those who reported not having any anal or vaginal sex in the past 3 months were then excluded, and the prevalence of any CLS, any CLS-D, and any CLS-C was assessed among the remaining MSM (who reported any sex in the past 3 months.) Associations were then examined between the mutually exclusive variable of sexual behaviour and sociodemographic, lifestyle, HIV-related factors and other sexual behaviours (among MSM who had anal or vaginal sex only). χ^2^ tests, χ^2^ tests for trend, or Kruskal-Wallis tests were used to compare across the four categories reporting anal or vaginal sex in the past 3 months only.

### Additional definitions of higher HIV risk CLS-D

We assessed the prevalence of various definitions of higher HIV risk CLS-D among all MSM with available data, by incorporating different criteria. This resulted in eight possible definitions of higher HIV risk CLS-D, which are defined explicitly in figure 3.

## Results

### ASTRA demographics

A total of 3258 HIV-diagnosed men and women completed the questionnaire (response rate 64%).^2^ This analysis reports on 2189 MSM diagnosed with HIV for ≥3 months prior to ASTRA, of whom 95% identified as gay, 5% as bisexual, and 90% as white; 44% had a university degree and 61% were employed. Mean age was 46 years (SD 9.4) and median time since HIV diagnosis was 10 years (IQR 5–16). Almost 87% were on ART, of whom 89% had clinic-recorded VL ≤50 c/mL at the last known test (93% had VL ≤200 c/mL). The timing of last available clinic VL result was ≤2 months before questionnaire (n=1088,50.0%), 2–4 months before (n=562,25.8%), 4–6 months before (n=338,15.5%), 6–12 months before (n=173,7.9%) and ≥12 months before (n=14,0.6%).

### Prevalence of sexual behaviours and association with other factors

Among all 2189 HIV-diagnosed MSM, overall prevalence estimates for sexual behaviour measures in the past 3 months were as follows: any anal or vaginal sex: 63.6% (95% CI 61.5% to 65.6%, n/N=1392/2189), any CLS (with HIV-serodifferent and/or HIV-seroconcordant partners): 38.2% (36.2% to 40.4%, 836/2189), any CLS-D: 16.3% (14.8% to 17.9%, 357/2189) and any CLS-C: 28.7% (26.8% to 30.6%, 628/2189).

When classifying all 2189 MSM according to the single variable of five mutually exclusive categories, the prevalence of sexual behaviours in the past 3 months was as follows: (1) higher HIV risk CLS-D: 4.2% (95% CI 3.5% to 5.2%, n=93), (2) other CLS-D: 12.1% (10.8% to 13.5%, n=264), (3) ‘CLS-C without CLS-D’: 21.9% (20.2% to 23.7%, n=479), (4) condom-protected sex only: 25.4% (23.6% to 27.3%, n=556) and (5) no anal or vaginal sex: 36.4% (34.3% to 38.4%, n=797).

A total of 1392 MSM reported having anal and/or vaginal sex in the previous 3 months; 1360 had anal sex with men only, 11 had anal or vaginal sex with women only and 21 had anal or vaginal sex with both men and women. The prevalence of any CLS in this group was 60.1% (57.5% to 62.6%, n/N=836/1392), that of any CLS-D was 25.6% (23.4% to 28.0%, 357/1392) and that of any CLS-C was 45.1% (42.5% to 47.7%, 628/1392).

Table 2 shows the associations of sociodemographic, lifestyle, and HIV-related factors with the mutually exclusive variable of sexual behaviour (n=2189 MSM). Compared with the four sexually active groups (1–4), men who did not have anal or vaginal sex in the previous 3 months (group 5) were older, diagnosed with HIV for longer, less likely to have a university degree or employment, more likely to be born in the UK, and less likely to have a stable partner. There were some differences in socio-demographic factors between the four sexually active groups in terms of age, length of time living with diagnosed HIV, financial hardship and stable partner status; MSM who had higher HIV risk CLS-D were more likely to be younger, to have been diagnosed for a shorter period of time, and to report financial hardship, compared with those who had other CLS-D, ‘CLS-C without CLS-D’, or condom-protected sex only. Men in the two CLS-D groups and those who reported condom-protected sex were much more likely than those in the CLS-C group to have an HIV-serodifferent stable partner. Conversely, those in the CLS-C group were more likely to have an HIV-positive stable partner. In terms of lifestyle factors, There were marked differences across the four groups in terms of lifestlye factors, MSM in the three CLS groups were substantially more likely to report recreational drug and polydrug use in the past 3 months compared with those who had condom-protected sex. The prevalence of harmful alcohol consumption was also significantly higher among MSM who had higher HIV risk CLS-D compared with the other three sexually active groups (p<0.05 for all above factors compared across groups 1 to 4). When excluding cannabis and nitrites from the definition of polydrug use, the prevalence of using ≥4 drugs in the past 3 months was highest among MSM who had ‘CLS-C without CLS-D’ (44.6%), followed by those who had higher HIV risk CLS-D (32.7%), other CLS-D (29.2%), and condom-protected sex (19.1%), and was lowest for those who did not have sex (15.9%). These patterns of associations were broadly similar after adjustment for age, time since HIV diagnosis, and stable partner status (data not shown).

**Table 2:** Sociodemographic, lifestyle and HIV-related factors associated with sexual behaviours in the past 3 months among MSM diagnosed with HIV for at least 3 months (n=2189)

			Sexual activity in the past 3 months (n=2189 MSM)								
	(1) Higher HIV risk CLS-D (n=93)		(2) Other CLS-D (n=264)		(3) CLS-C without CLS-D (n=479)		(4) Condom-protected sex only (n=556)		(5) No anal or vaginal sex (n=797)		p-value across groups 1–4 only
Mean age (SD) (N=2135)	42	(9.0)	46	(9.2)	43	(8.8)	45	(9.6)	49	(9.0)	0.020^ANOVA^
Median years since HIV diagnosis (IQR) (N=2153)	5	(2–10)	10	(6–15)	8	(4–14)	9	(4–15)	12	(7–18)	<0.001^KW^
	**n**	**%**	**n**	**%**	**n**	**%**	**n**	**%**	**n**	**%**	
Ethnicity (N=2154)											
White	80	87.9	230	88.8	428	90.3	477	87.5	713	90.8	
Black, Asian, Mixed, Chinese or other	11	12.1	29	11.2	46	9.7	68	12.5	72	9.2	0.568
Place of birth (N=2189)											
UK	60	64.5	181	68.6	328	68.5	357	64.2	576	72.3	
Outside the UK	33	35.5	83	31.4	151	31.5	199	35.8	221	27.7	0.421
Education (N=2149)											
None or up to A levels	54	60.0	138	53.1	245	51.7	292	53.4	470	60.4	
University degree or above	36	40.0	122	46.9	229	48.3	255	46.6	308	39.6	0.549
Employment (N=2154)											
Employed full or part-time	59	64.8	167	64.2	331	69.7	370	67.8	391	50.0	
Unemployed or other (student, retired and carer)	32	35.2	93	35.8	144	30.3	176	32.2	391	50.0	0.455
Money for basic needs (N=2158)											
Always	41	45.1	135	52.1	254	53.5	313	57.3	371	47.1	
Mostly or sometimes	35	38.5	102	39.4	193	40.6	196	35.9	345	43.8	
No	15	16.5	22	8.5	28	5.9	37	6.8	71	9.0	0.012(T)
Social support* (N=2170)											
Highest	29	31.2	82	31.2	158	33.3	198	35.7	217	27.6	
Medium	50	53.8	152	57.8	265	55.8	303	54.7	458	58.3	
Lowest	14	15.1	29	11.0	52	10.9	53	9.6	110	14.0	0.467(T)
Stable partner HIV serostatus (N=2189)											
No stable partner	45	48.4	108	40.9	172	35.9	241	43.3	419	52.6	
HIV-positive stable partner	15	16.1	41	15.5	253	52.8	80	14.4	121	15.2	
HIV-serodifferent status stable partner	33	35.5	115	43.6	54	11.3	235	42.3	257	32.2	<0.001
Recreational drug use in past 3 months (N=2189)											
No	23	24.7	90	34.1	146	30.5	264	47.5	555	69.6	
Yes	70	75.3	174	65.9	333	69.5	292	52.5	242	30.4	<0.001
Polydrug use in past 3 months (N=2189)											
0–3 drugs	68	73.1	192	72.7	320	66.8	496	89.2	767	96.2	
≥4 drugs	25	26.9	72	27.3	159	33.2	60	10.8	30	3.8	<0.001
Possible harmful/hazardous alcohol consumption† (N=2189)											
No	67	72.0	211	79.9	406	84.8	466	83.8	673	84.4	
Yes	26	28.0	53	20.1	73	15.2	90	16.2	124	15.6	0.014
ART status (N=2178)											
On ART	35	38.0	263	100.0	395	82.6	472	85.2	723	91.4	
Not on ART	57	62.0	0	-	83	17.4	82	14.8	68	8.6	–
Clinic-recorded viral load‡ (N=2175)											
>50 c/mL	90	96.8	0	-	127	26.6	130	23.5	134	17.0	*–*
>200 c/mL	72	77.4	0	-	107	22.4	103	18.7	104	13.2	

p Values by χ^2^ test unless otherwise specified (analysis of variance (ANOVA), Kruskal-Wallis rank test (KW), χ^2^ test for linear trend (T)); CLS, condomless sex; CLS-D, CLS with HIV-serodifferent (HIV-negative or HIV-unknown status) partners; higher HIV risk CLS-D, CLS-D plus either not being on antiretroviral therapy (ART) or having latest clinic-recorded viral load >50 c/mL; CLS-C, CLS with HIV-seroconcordant (other HIV-positive) partners; p Values for ART status and viral load comparisons not shown as these differ by definition across groups of sexual behaviour in the past 3 months.

*Duke-UNC Functional Social Support Questionnaire scores: 21–25, highest; 13–20, medium; and 0–12, lowest.

†Score≥6 on modified WHO AUDIT-C (first two questions only).

‡Latest value available to participant at the time of the questionnaire.

MSM, men who have sex with men

### Number of sexual partners

**Figure 1:**
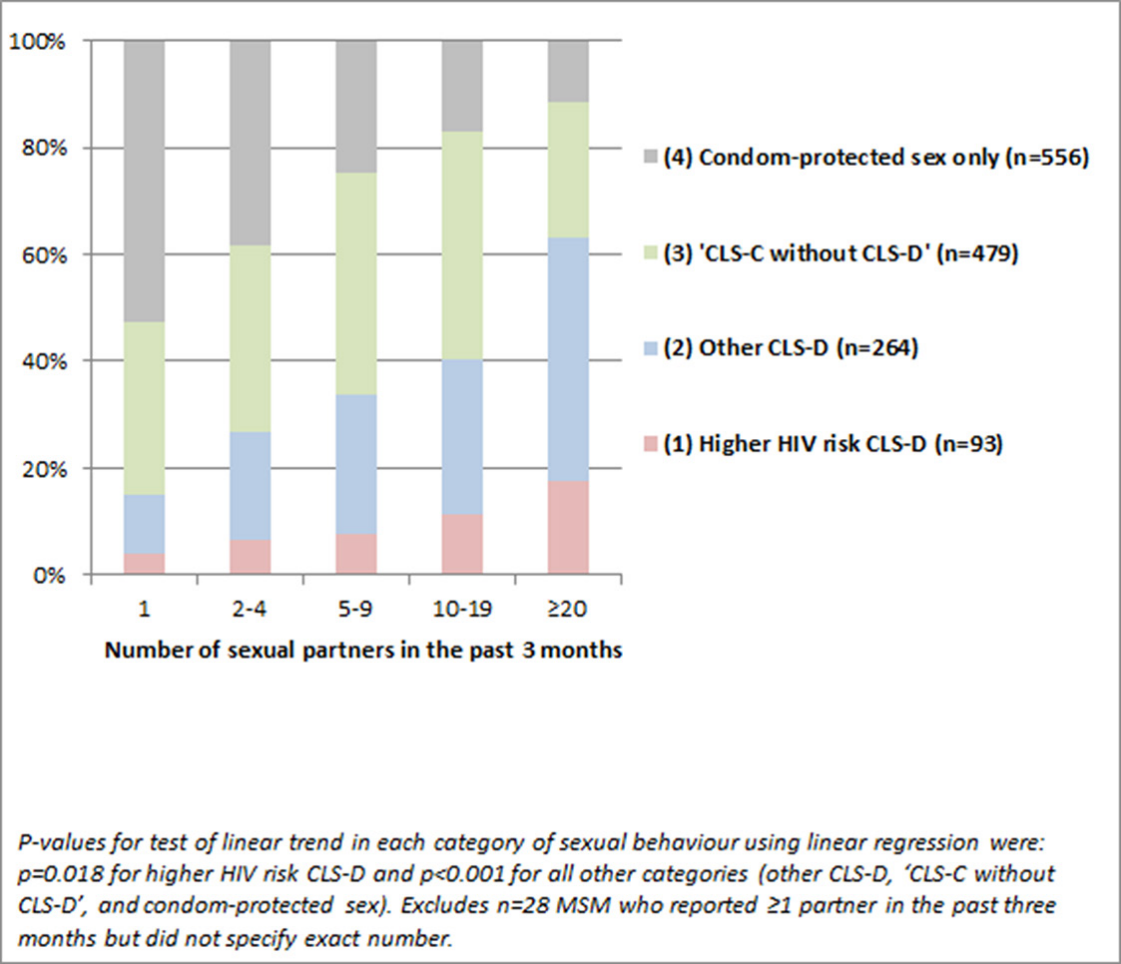
Distribution of sexual behaviour according to number of total sexual partners in the past 3 months (n=1392 HIV-diagnosed MSM reporting any anal or vaginal sex in the past 3 months). CLS, condomless sex; CLS-C, CLS with HIV-seroconcordant; CLS-D, CLS with HIV-serodifferent partners.

Figure 1 shows the distribution of the mutually exclusive sexual behaviour variable (groups 1 to 4 only) according to the total number of sexual partners participants reported in the past 3 months (n=1392 MSM who had anal or vaginal sex in the past 3 months, of whom 1357 had information on number of sexual partners). Prevalence of higher HIV risk CLS-D and other CLS-D increased substantially with higher total number of partners in the past 3 months.

Information on number of CLS-D partners was available for 320 (of 357) MSM who had CLS-D in the past 3 months; of the 84 MSM who had higher HIV risk CLS-D, 52.4% had only one CLS-D partner, 31.0% had two to four CLS-D partners, and 16.7% had five or more CLS-D partners; the corresponding proportions for 236 MSM who had ‘other CLS-D’ were 57.6%, 27.1%, and 15.2%. Information on number of CLS-C partners was available for 476 (of 479) MSM who had ‘CLS-C without CLS-D’; 59.4% had one CLS-C partner, 24.6% had two to four CLS-C partners and 16.0% had five or more CLS-C partners in the past 3 months.

### Other sexual behaviours, STIs and attitudes

Among 236 MSM who reported any other STI diagnosis in the past 3 months, 73.3% had one STI and 26.7% had two or more STIs. The majority (58.4%) of 310 responses were for bacterial STIs (syphilis, gonorrhoea, chlamydia, and LGV), 11.9% were for new or recurrent genital herpes, 9.9% were for new or recurrent genital warts, 7.1% were for new hepatitis B or C diagnosis and the remaining were for NSU/NGU and other (unspecified) STIs.

**Figure 2:**
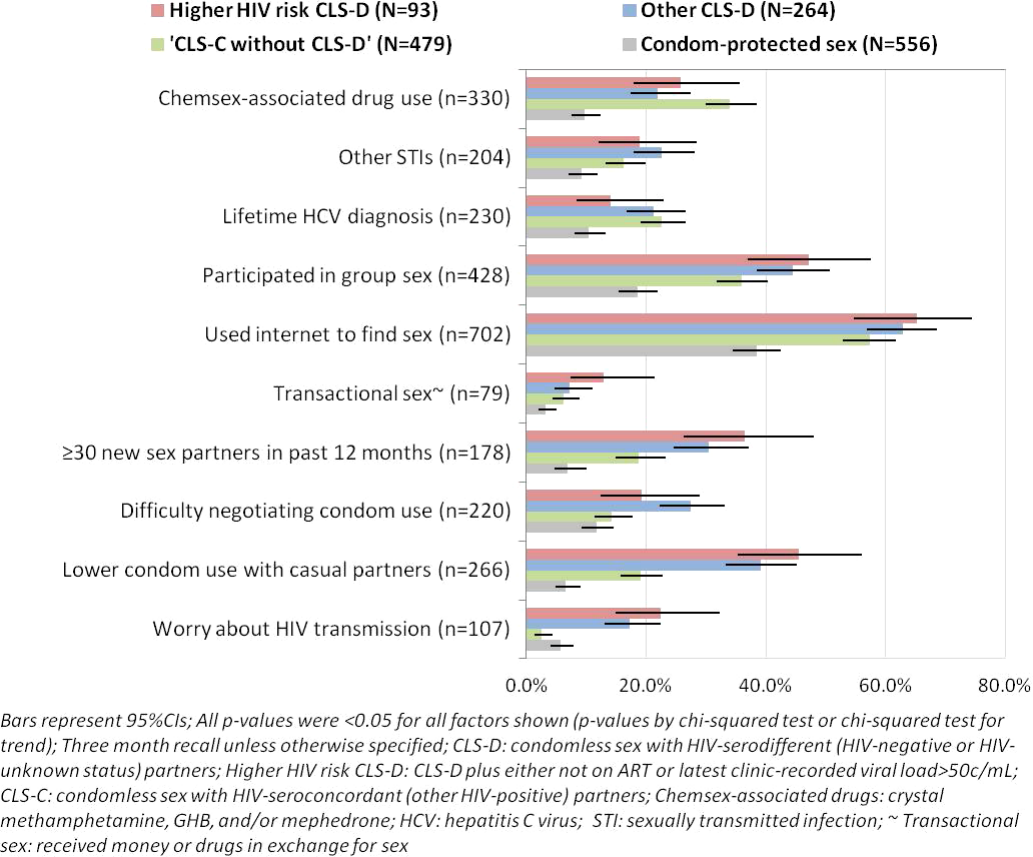
Associations between other sexual behaviours, STIs, attitudes to condom use and single variable of sexual behaviour in the past 3 months (n=1392 HIV-diagnosed MSM reporting any anal or vaginal sex in the past 3 months).

Figure 2 shows the prevalence (and 95% CIs) of other sexual behaviours, STIs, attitudes towards condom use and HIV transmission, among 1392 MSM who reported any anal or vaginal sex only. Substantial differences were observed between the four sexually active groups on all sexual behaviours, STIs and attitudes. Compared with MSM who had CLS (groups 1–3), those who had condom-protected sex (group 4) had much lower prevalence of chemsex-associated drug use, STIs, group sex, high partner numbers,  and use of the internet to find sex, and were less likely to report difficulty in using condoms and worries about HIV transmission. The prevalence of high partner numbers, group sex, use of the internet to find sex, and reporting transactional sex in the past 3 months was highest among men having higher HIV risk CLS-D (group 1) and those having other CLS-D (group 2), followed by those who had ‘CLS-C without CLS-D’ (group 3). Men reporting any CLS-D (groups 1 and 2) were also more likely than the other two sexually active groups to report difficulty in discussing or using condoms with new partners. Almost 23% of MSM who had higher HIV risk CLS-D and 17.3% of those who had other CLS-D were worried they could have transmitted HIV recently. Prevalence of chemsex-associated drug use in the past 3 months and of lifetime hepatitis C diagnosis was highest for MSM who had ‘CLS-C without CLS-D’ (group 3). These patterns of associations were similar after adjustment for age, time since HIV diagnosis, and stable partner status (data not shown).

### HIV risk reduction sexual behaviours during CLS-D

The prevalence of sexual positioning and withdrawal prior to ejaculation was assessed among 314 MSM who reported any CLS-D (higher HIV risk or other CLS-D); 28.0% (95%CI 23.3% to 33.3%, n=88) reported being the insertive partner and ejaculating inside, 29.3% (24.5% to 34.6%, n=92) reported  being insertive but never ejaculating inside, 40.1% (34.9% to 45.7%, n=126) reported always being the receptive partner, and 2.5% (1.3% to 5.1%, n=8) reported having CLS-D with women only in the past 3 months. There were no significant differences in the prevalence of sexual positioning or ejaculation between MSM who reported higher HIV risk CLS-D and those who reported other CLS-D (insertive with ejaculation: 24.1% vs 29.4%, respectively; insertive without ejaculation: 25.3% vs 30.7%; receptive only: 48.2% vs 37.2%, Fisher’s exact test, p=0.392).

### Additional definitions of higher HIV risk CLS-D

**Figure 3:**
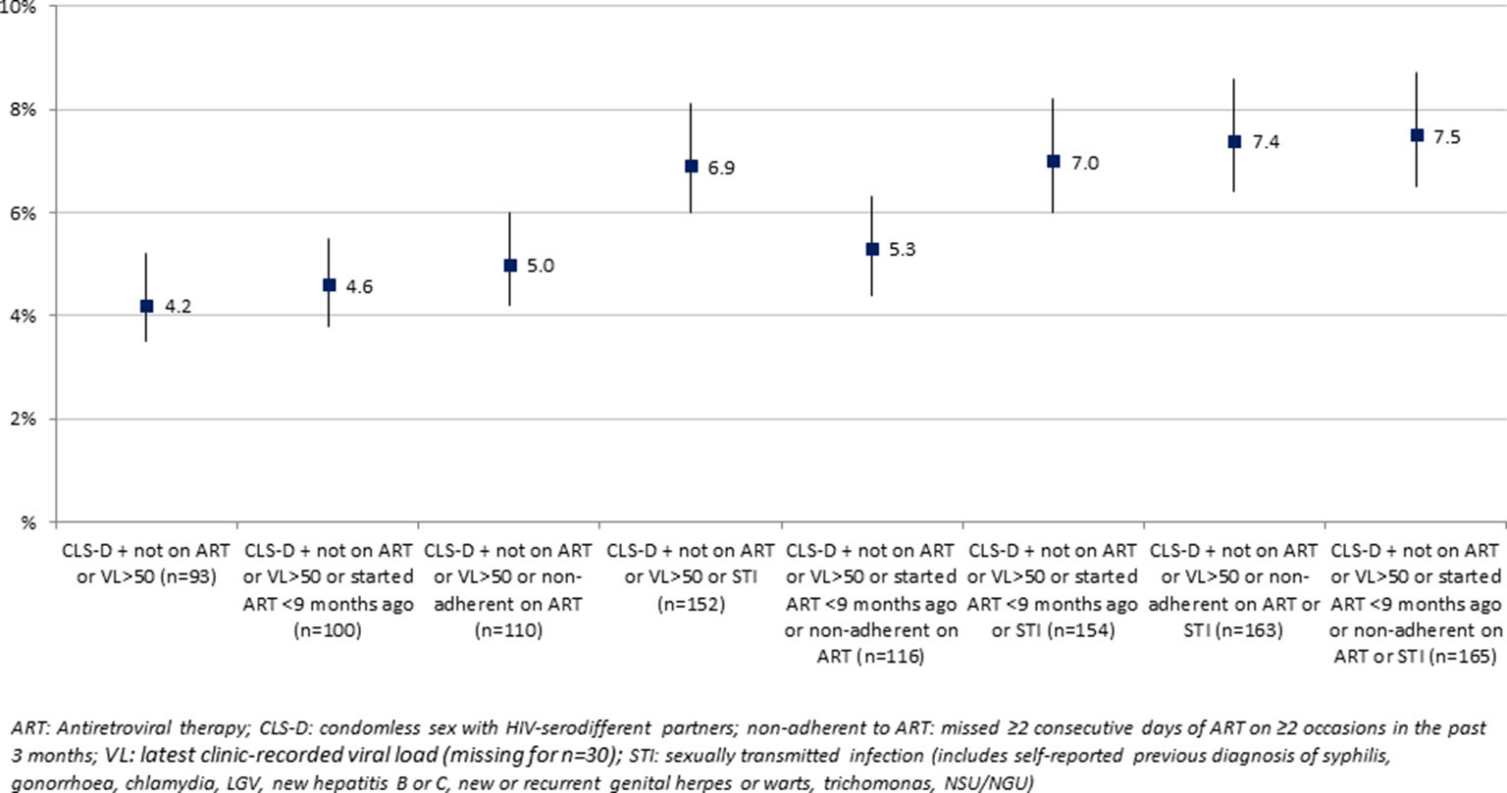
Prevalence (95% CI) of higher HIV transmission risk CLS-D among 2189 HIV-diagnosed MSM according to criteria incorporated (ART status, viral load level, time since started ART, ART non-adherence, and self-reported other STI diagnosis in past 3 months).

Figure 3 shows additional definitions for higher HIV risk CLS-D in the previous 3 months, according to various criteria incorporated. The main definition required reporting CLS-D in the previous 3 months with at least one of two additional factors: not on ART or clinic-recorded VL >50 c/mL. In further definitions, the set of additional factors that could confer higher HIV risk CLS-D was widened to include the following: started ART months ago, non-adherence to ART, and other diagnosed STIs in the past 3 months. The prevalence of each definition of higher HIV risk CLS-D in the previous 3 months was assessed among 2189 HIV-diagnosed MSM, and  ranged from 4.2% to 7.5% depending on criteria included. For example, 7.5% of all HIV-diagnosed MSM were classified as having higher HIV risk CLS-D if they reported having CLS-D in the previous 3 months and either were not on ART or had latest VL >50 c/mL or started ART <9 months ago or reported non-adherence to ART or another diagnosed STI. Compared with the prevalence of any CLS-D in the past 3 months (16.3% of 2189 MSM), the prevalence of higher HIV risk CLS-D was lower by 53%–74% in relative terms. Results were similar when VL cut-off was increased to >200 c/mL (prevalence ranged from 3.5% to 7.1%).

## Discussion

In this large multicentre UK study of HIV-diagnosed MSM, over a third reported having CLS in the previous 3 months: 16% had CLS with HIV-negative or unknown status partners (CLS-D) and 22% had CLS with HIV-seroconcordant partners only (‘CLS-C without CLS-D’). Only 4.2% of MSM had CLS-D while either not on ART or with detectable VL, and they were, therefore, classified as having CLS-D with higher risk of HIV transmission. Compared with men who reported condom-protected sex only, MSM who reported any type of CLS in the past 3 months had higher prevalence of STIs, recreational and chemsex-associated drug use, group sex, higher partner numbers, and lifetime hepatitis C diagnosis.

Similar prevalence estimates of CLS (CLS-D and CLS-C) have been observed in studies of HIV-diagnosed MSM recruited from HIV-outpatient clinics in the UK^21–25^ and the USA.^26–29^ Our results are also in line with baseline data from the START trial of early versus deferred ART initiation; CLS-D in the past 2 months was 15.1% among ART-naive HIV-diagnosed MSM in the European sample (n/N=177/1172), and 12.1% when excluding MSM diagnosed in the past 3 months (as in this ASTRA analysis).^30^ CLS with other HIV-positive partners only was also prevalent in our study, suggesting actual or perceived serosorting (selecting sexual partners based on HIV status). The consistent prevalence of CLS estimates between the aforementioned clinic-based studies and ASTRA is encouraging in supporting our ability to reliably and repeatedly measure such behaviours and capture trends. These results also suggest that there has not been an increase in the prevalence of CLS-D among HIV-diagnosed MSM in the UK during the past decade. CLS with HIV-serodifferent partners (CLS-D) denoted high-risk sex for HIV transmission prior to the emergence of conclusive evidence on the extremely low risk of HIV transmission when the HIV-diagnosed partner is on suppressive ART.^11 12^ Coupled with changes in treatment guidelines recommending ART initiation at any CD4 count,^31^ these important results may affect trends in CLS among HIV-diagnosed MSM. In ASTRA, the prevalence of higher HIV risk CLS-D in the past 3 months was overall low, ranging from 4.2% when considering CLS-D while not on ART or with detectable VL, to 7.5% when additionally considering time since started ART, non-adherence, or other STI co-infections. There is no other evidence to date on the prevalence of higher HIV risk CLS with HIV-serodifferent partners among HIV-diagnosed MSM in the UK. Recently, a number of US studies have estimated the prevalence of CLS-D when VL is not suppressed among HIV-diagnosed MSM outpatients. In the previous 3 months, estimates of higher HIV risk CLS-D were as follows: 18.9% in the Adolescent Medicine Trials Network (n=991 MSM, defined as CLS-D and latest clinic-recorded VL ≥200 c/mL)^w1^ and 34% in the international HPTN063 cohort study (n=200, defined as CLS-D and ‘detectable VL’ or other STI)^w2^. In the previous 6 months, estimates of higher HIV risk CLS-D were as follows: 3.4% in the HIV Outpatient Study (n=902, defined as insertive CLS-D and VL ≥400 c/mL)^w3^ and 34% in the baseline survey of Fenway Health (n=201, CLS-D and either VL >75 c/mL or diagnosis of gonorrhoea, syphilis, or chlamydia in the past year)^w4^. Finally, in the past year, prevalence of CLS-D with higher HIV transmission risk was 6.0% in the US nationally representative Medical Monitoring Project (n=1897, defined as CLS-D and one or more VL ≥400 c/mL in the past 12 months)^w5^. In ASTRA, when additionally including sexual positioning in the definition, the prevalence of higher HIV risk CLS-D (defined as insertive CLS-D and either not on ART or latest clinic VL ≥50 c/mL) was 1.8% (95% CI 1.4% to 2.5%). The disparity in estimates is due to differences in definitions, study design and methodology, and method of survey administration as well as potential genuine differences across population/demographic groups. In particular, study eligibility criteria varied, as certain studies required participants to be diagnosed for at least 12 months^w6 w7^, which may have underestimated the prevalence of higher HIV risk CLS-D by excluding people who were not on stable ART. Conversely, other studies excluded participants who did not report CLS ^w2 w4^ leading to higher prevalence estimates.

It is still unclear which factors, additional to ART use and VL level, should be incorporated in clinical and epidemiological definitions of sex with higher risk of HIV transmission, particularly in the context of CLS with multiple HIV-serodifferent partners. There is also a need for better understanding of the effect of different levels of non-adherence on the likelihood of viral rebound to a level that will significantly impact HIV transmission risk. Additional longer-term follow-up from the PARTNER study of HIV-serodifferent couples^12^ will also be crucial in providing precise estimates of HIV transmission risk among MSM in the context of effective ART. Such information is important in refining current guidelines and in helping HIV-diagnosed individuals and their partners make informed decisions on having CLS-D safely. Standardised definitions of higher HIV risk CLS-D would be useful for epidemiological studies of behavioural surveillance.

A substantial proportion of men engaging in higher HIV risk CLS-D (and in other CLS-D) in ASTRA reported chemsex-associated drug use, group sex, and high numbers of sexual partners. A strong association was also observed between the total number of sexual partners in the past 3 months and prevalence of CLS and CLS-D in the past 3 months. This may emphasise the challenge of maintaining consistent condom use and accurately ascertaining partners’ HIV serostatus in the context of high partner numbers. Consistent with other studies^12 26^ ^w4^, results from ASTRA may indicate use of perceived risk reduction strategies during CLS-D, such as being the receptive partner and withdrawal before ejaculation.

While it is encouraging that a sizeable proportion of HIV-diagnosed MSM in ASTRA restrict CLS to HIV-positive partners only, this does not eliminate the risk of other STIs. In fact, MSM who had ‘CLS-C without CLS-D’ had the highest prevalence of lifetime hepatitis C diagnosis and of chemsex-associated drug use. During the past decade, increased diagnoses of other STIs among HIV-diagnosed MSM in the UK have coincided with the emergence of sexually transmissible enteric infections^w7^. These overlapping epidemics suggest that sexual networks of HIV-diagnosed MSM engaging in serosorting may contribute to transmission of other STIs in the UK. In terms of assessing and monitoring risk of transmission of STIs among HIV-diagnosed MSM, ‘any condomless sex’ is likely to be the most relevant measure.

ASTRA is the largest questionnaire study of HIV-diagnosed individuals in the UK to date; the study population can be considered representative of HIV-diagnosed MSM in the UK, as access to healthcare is universal and over 95% of HIV-diagnosed people access specialist HIV services. The response rate (64%) is satisfactory and comparable to earlier similar studies.^22 23 25^ There were no significant differences in VL or CD4 cell count between responders and those who did not respond but consented to participate.^2^ Our study does have some limitations. Self-reported sexual behaviour may be subject to error and bias; underreporting of CLS is possible and may have led to underestimation of prevalence. A single clinic-recorded plasma VL measure was used in the definition of higher HIV risk CLS-D. Although this measure was the latest VL result available to the participant, it occurred at a variable time in relation to the 3-month recall period for sexual behaviour; VL status may have changed over this period for some participants. The length of time with viral suppression specifically could not be incorporated. Among HIV-diagnosed MSM who had CLS with other HIV-positive partners only, it was not possible to ascertain whether a partner’s HIV-positive serostatus was assumed or known with confidence.

Although ASTRA was conducted after the 2008 Swiss statement, when expert opinion on reduced risk of HIV transmission with suppressed VL was widely publicised, it was conducted prior to publication of results from HPT052^11^ and PARTNER.^12^ The sexual behaviour of HIV-diagnosed MSM, as well as the prevalence of higher HIV risk CLS-D, may change with increasing awareness of results from these studies.

## Conclusions

In conclusion, among HIV-diagnosed MSM in the UK, the prevalence of CLS in the previous 3 months was relatively high, but in line with results from similar UK studies in the past decade. The high prevalence of CLS with other HIV-positive partners may indicate active serosorting and warrants further attention as transmission of other STIs is high among people with HIV. Although 1 in 6 HIV-diagnosed MSM reported having CLS with HIV-serodifferent partners (CLS-D), <1 in 14 reported CLS-D with an appreciable risk of HIV transmission. As ART use expands, it remains crucial to promote sustained high ART adherence, regular VL monitoring and ongoing awareness of personal VL level^w8 w9^. In order for behavioural studies on HIV transmission to be representative of the developments in understanding of HIV transmission and prevention, there is a need to move away from definitions of ‘unsafe’ or ‘risky’ sex based solely on CLS with HIV-serodifferent partners among people with diagnosed HIV. Incorporating ART status, latest VL, and other factors that impact strongly on HIV transmission will ensure research measures are based on contemporary evidence.

**Key messages**

- In this large UK study of HIV-diagnosed men who have sex with men (MSM) outpatients, over a third had any condomless sex (CLS), 22% had CLS with HIV-seroconcordant partners only and 17% had any CLS with HIV-serodifferent partners (CLS-D).
- CLS-D with an appreciable risk of HIV transmission (accounting for detectable viral load) occurred in a small minority of HIV-diagnosed MSM (4.2%).
- While ‘any CLS’ is still a useful measure of other STI transmission risk behaviour, CLS-D is no longer the most appropriate measure of HIV transmission risk behaviour; accounting for viral load is important.

10.1136/sextrans-2016-053029.supp1 Supplementary Appendix 1
