# Supplementary material for: Condomless sex in HIV-diagnosed men who have sex with men in the UK: prevalence, correlates, and implications for HIV transmission
Source: Sex Transm Infect. 2017 Jul 5;93(8):590–8. doi: 10.1136/sextrans-2016-053029 (PMC5739863; doi:10.1136/sextrans-2016-053029)
Supplement: Supplementary Appendix 1 [file sextrans-2016-053029supp001.docx]

# Web appendix

w1. Wilson PA, Kahana SY, Fernandez MI, Harper GW, Mayer K, Wilson CM, et al. Sexual Risk Behavior Among Virologically Detectable Human Immunodeficiency Virus-Infected Young Men Who Have Sex With Men. JAMA Pediatr. 2016 Feb;170(2):125–31.

w2. Magidson JF, Li X, Mimiaga MJ, Moore AT, Srithanaviboonchai K, Friedman RK, et al. Antiretroviral Medication Adherence and Amplified HIV Transmission Risk Among Sexually Active HIV-Infected Individuals in Three Diverse International Settings. AIDS Behav. 2016 Apr;20(4):699–709.

w3. Durham MD, Buchacz K, Richardson J, Yang D, Wood K, Yangco B, et al. Sexual risk behavior and viremia among men who have sex with men in the HIV Outpatient Study, United States, 2007-2010. J Acquir Immune Defic Syndr. 2013 Jul 1;63(3):372–8.

w4. Mayer KH, Skeer MR, O’Cleirigh C, Goshe BM, Safren SA. Factors associated with amplified HIV transmission behavior among American men who have sex with men engaged in care: implications for clinical providers. Ann Behav Med. 2014 Apr;47(2):165–71.

w5. Mattson CL, Freedman M, Fagan JL, Frazier EL, Beer L, Huang P, et al. Sexual risk behaviour and viral suppression among HIV-infected adults receiving medical care in the United States. AIDS. 2014 May 15;28(8):1203–11.

w6. Suzan-Monti M, Lorente N, Demoulin B, Marcellin F, Préau M, Dray-Spira R, et al. Sexual risk behaviour among people living with HIV according to the biomedical risk of transmission: results from the ANRS-VESPA2 survey. J Int AIDS Soc. 2016 Jan;19(1):20095.

w7. Simms I, Field N, Jenkins C, Childs T, Gilbart V, Dallman T, et al. Intensified shigellosis epidemic associated with sexual transmission in men who have sex with men - Shigella flexneri and S. sonnei in England, 2004 to end of February 2015. Eurosurveillance. 2015;20(15):pii=21097.

w8. Lampe F. Sexual behaviour among people with HIV according to self-reported antiretroviral and viral load status. Results from the ASTRA study. AIDS. 2016 Mar 31;30(11):1745–59.

w9. Sewell J, Daskalopoulou M, Nakagawa F, Lampe F, Edwards S, Perry N, et al. Accuracy of self-report of HIV viral load among people with HIV on antiretroviral treatment. HIV Med. 2016 Dec 22;
